# Impact of Exercise Intervention Combined with Optimal Mediterranean Diet Adherence during Pregnancy on Postpartum Body Composition: A Quasi-Experimental Study—The GESTAFIT Project

**DOI:** 10.3390/nu15204413

**Published:** 2023-10-18

**Authors:** Marta Flor-Alemany, Pedro Acosta-Manzano, Jairo H. Migueles, Pontus Henriksson, Marie Löf, Virginia A. Aparicio

**Affiliations:** 1 Department of Physiology, University of Granada, 18071 Granada, Spain; floralemany@ugr.es (M.F.-A.); virginiaparicio@ugr.es (V.A.A.); 2 Biomedical Research Centre (CIBM), Institute of Nutrition and Food Technology (INYTA), University of Granada, 18016 Granada, Spain; 3 Sport and Health University Research Institute (IMUDS), 18007 Granada, Spain; acostapedro23@gmail.com; 4 Institute of Human Movement Science, Sport and Health, University of Graz, 8010 Graz, Austria; 5 PROFITH “Promoting FITness and Health through Physical Activity” Research Group, Department of Physical Education and Sports, Faculty of Sport Sciences, University of Granada, 18071 Granada, Spain; 6 Department of Health, Medicine and Caring Sciences, Linköping University, 58183 Linköping, Sweden; pontus.henriksson@liu.se; 7 Department of Biosciences and Nutrition, Karolinska Institutet, 14152 Huddinge, Sweden; marie.lof@ki.se

**Keywords:** nutrition, lifestyle behaviors, dietary patterns, gestation, postpartum weight retention

## Abstract

This study aimed to investigate whether the effects of an exercise program during pregnancy on postpartum body composition are moderated by following a healthy dietary pattern (i.e., Mediterranean diet (MD)). Eighty-three pregnant women (control n = 40, exercise n = 43) were included in the present quasi-experimental study. The exercise intervention consisted of a 60 min, 3 day/week throughout pregnancy from gestational week 17, supervised concurrent (aerobic + resistance) exercise program. A food frequency questionnaire and the MD Score (min–max: 0–50) were employed to assess dietary habits and the MD adherence during pregnancy, respectively. Postpartum body composition was measured with dual-energy X-ray absorptiometry, 6 weeks postpartum. The body mass index and the gynecoid fat mass at postpartum were lower in the exercise compared to the control group (*p* = 0.018 and *p* = 0.047, respectively). There was an interaction showing that the MD adherence during pregnancy positively moderated the effects of the exercise intervention on postpartum lean mass (*p* = 0.024), fat mass percentage (*p* = 0.092), android fat mass (*p* = 0.076), and android-to-gynecoid fat mass (*p* = 0.019). The Johnson–Neyman technique revealed that the effects of exercise were enhanced at a MD score of ~31 for lean mass, ~25 for fat mass, ~23 for android fat mass and ~29 for android-to-gynecoid fat mass. Our results suggest that a concurrent-exercise training plus an optimal MD adherence during pregnancy might be a useful strategy to promote a healthier body composition at the postpartum period.

## 1. Introduction

Pregnancy can trigger physiological permanent changes in body composition and weight gain [1]. In Europe, 51% of women experience excessive gestational weight gain (GWG) [2], which is often retained long term after birth (postpartum weight retention (PPWR)) [3,4,5], contributing to the obesity rates in adult women [4,6]. PPWR is particularly harmful as it promotes central rather than peripheral fat accumulation [1,7,8]. Overall and central/visceral excess of fat are well-known risk factors for greater cardiometabolic risk (e.g., hypertension, impaired glucose tolerance, and elevated triglycerides) [9,10]. Hence, it is imperative to find effective strategies to improve maternal body composition at postpartum to avoid future complications.

Exercise interventions during pregnancy can potentially control and avoid excessive GWG and PPWR [11,12,13]. The current scientific literature is limited on the effects of different types of exercise programs on GWG, and specially on PPWR and postpartum body composition. Some studies have shown improvements in GWG and body composition as a result of aerobic training alone [13], or in combination with resistance training during pregnancy [12,14] or postpartum [15], while another study did not find any effect on GWG, PPWR, or fat measurements [16].

Regarding the diet, two previous studies have examined the relationship between dietary energy intake and PPWR [4,17]. This relationship might be further investigated by examining the dietary patterns beyond the energy intake. Dietary patterns can capture interactions between individual dietary components [18]. The Mediterranean Diet (MD), characterized by high intake of vegetables, fruits, pulses, fish, olive oil, cereals, nuts, and seeds, and a low consumption of processed food, red meat and dairy products [19], promotes healthy weight in adults [20], and lowers cardiometabolic risk in pregnant women [21]. Moreover, a high MD adherence has been associated with greater fat-free mass [22], lower body mass index (BMI), and weight gains [23,24] in non-pregnant adults. However, to the best of our knowledge, no previous study has explored the relationship between MD adherence during pregnancy and postpartum body composition.

Overall, interventions including diet and exercise components appear to be more effective in promoting postpartum weight and fat mass loss [25], and preserving lean body mass, compared to diet [26] or exercise alone [16]. Specifically, the GESTAFIT project shown that exercise during gestation may help optimize GWG and PPWR [27] but a deeper study of the body composition will provide clinical meaningful information. In fact, according to a systematic review [26], exercise should be complemented with a healthy diet to promote weight loss at postpartum. Thus, it is necessary to study the interaction effect of an exercise intervention throughout gestation with a healthy nutritional pattern. Therefore, this study aimed (1) to investigate the effects of an exercise intervention delivered to pregnant women on postpartum body composition and (2) to examine whether an optimal MD adherence during pregnancy modulates these effects.

## 2. Materials and Methods

### 2.1. Study Design and Participants

We employed data from the GESTAFIT project (identifier: NCT02582567) [28]. The study was carried out at the “Sport and Health University Research Institute” (Granada, Spain), and at the “San Cecilio and Virgen de las Nieves University Hospitals” from November 2015 to April 2018. This project was approved by the Clinical Research Ethics Committee of Granada, Government of Andalusia, Spain (code: GESFIT-0448-N-15). The complete methodology of this project, and the inclusion–exclusion criteria (Supplementary Table S1) are available elsewhere [28]. We included women aged 20–40 years with a normal pregnancy course, and giving birth (singleton) at 37–42 weeks via spontaneous/vaginal delivery, or caesarean section without severe maternofoetal pathology. Of 384 pregnant women assessed for eligibility, 159 women were randomized. All women provided written informed consent. A total of 102 pregnant women who had valid data in postpartum body composition measurements and dietary habits were included in this quasi-experimental study. Among them, 83 participants were considered for the per-protocol intervention analyses (exercise n = 40, control n = 43) (Supplementary Figure S1). These are secondary analyses from the GESTAFIT Project [28]. Power calculations were determined based on the primary outcomes (GWG), and it was 52 women (26 per group) [28]. Additionally, a posteriori power calculation analyses showed that this study has a power of 80% to detect medium-sized effect sizes with the 83 women included in the analyses (f^2^ ≥ 0.09) [29].

### 2.2. Randomization and Blinding

The study was conducted in three waves. The GESTAFIT project was initially designed as a randomised control trial (computer-generated simple randomization). Nonetheless, the randomized component was broken in the second and third waves to ensure sufficient adherence to the program, which represents a frequent methodological barrier in antenatal exercise research [30]. Thus, half the women were not randomized but allocated to the control/exercise group according to their personal convenience. Most personnel were blinded to their allocation into the control/exercise group, excepting those responsible for the training sessions. 

### 2.3. Exercise Intervention

The exercise intervention consisted of a concurrent supervised tailored exercise program (from week 17 until delivery, 3 days/week, 60 min/session) of aerobic and resistance exercises of moderate-to-vigorous (mostly moderate with peaks of vigorous) intensity. Sessions consisted of a 10 min warm-up, a 40 min muscular (circuits of resistance exercises and short aerobic blocks) or aerobic block (dance or functional circuits), and a 10 min cooldown. Resistance exercises involved anterior and posterior chain dominant, pull, push, and core exercises. The exercise training program was designed following the standards by the American College of Obstetricians and Gynecologists [31] and the latest scientific evidence [32,33]. During the intervention, women were provided with 7 seminars to promote healthier pregnancies (File S1). 

### 2.4. Control Group

Pregnant women in the control group did not attend the exercise sessions and were asked to keep their usual activities, yet they were invited to the seminars at a different timing from the exercise group to avoid contamination.

### 2.5. Sociodemographics 

Sociodemographic characteristics of the study sample were gathered through medical files and questionnaires at the 16th g.w. (i.e., age, educational and marital status, and smoking). 

### 2.6. Maternal Anthropometry, Postpartum Weight Retention and Body Composition

Pre-pregnancy body weight was self-reported at recruitment (12th g.w.). Although measured weight is preferable, self-report is a cost-effective and practical measurement approach that shows very good concordance with measured body weight [34]. Weight was measured 6 weeks after giving birth (no shoes, light clothes) with an electronic scale (InBody-R20; Biospace, Seoul, Republic of Korea). Height was measured using a stadiometer (Seca 22, Hamburg, Germany). BMI was calculated as kg/m^2^. 

Postpartum body composition measurements were assessed using dual-energy X-ray absorptiometry (Discovery DXA system; Hologic, Marlborough MA, USA). Body composition outcomes included total lean mass and fat mass, fat mass percentage, android fat mass (truncal adiposity), gynecoid fat mass (peripheral fat), visceral fat, ratio of gynecoid to total fat mass, and ratio of android to gynoid fat mass. A whole-body scan was performed considering the manufacturer’s guidelines to ensure the quality of the data. The APEX 4.0.2. software (Hologic Series Discovery, Hologic, Marlborough, MA, USA) was used to draw an automatic delineation of anatomic regions. 

### 2.7. Physical Activity

Physical activity was monitored for 9 days (24 h/day, except for water activities) with non-dominant-wrist-worn accelerometers (ActiGraph GT3X+, Pensacola, FL, USA). Total and moderate-vigorous physical activity (min/day) were estimated the week before to the beginning of the intervention (at the 16th g.w.). 

### 2.8. Dietary Assessment and Mediterranean Diet Adherence

A food frequency questionnaire validated in Spanish adults [35] was administered by a trained nutritionist at the 16th g.w. and 34th g.w. to assess dietary habits. Women were asked about the frequency of consumption of the different food groups (never or number of times per day, week, month, or year). This study targeted women in the second trimester of pregnancy (13th to 27th g.w.). The first trimester is characterised by morning sickness, whereas dietary habits during the second trimester are more constant and representative of diet across the entire gestation [36]. Moreover, we observed similar MD adherence between the 16th g.w. and the 34th g.w. in our sample [37]. Consequently, the dietary habits of the 16th g.w. were considered for analyses. 

The MD Score developed by Panagiotakos et al. [19] was derived from the food frequency reports [35] to assess MD adherence as previously performed in this study sample [21]. The MD Score consists of eleven variables (wholegrain cereals, potatoes, fruits, vegetables, pulses, fish, olive oil, red wine, red meat and subproducts, poultry, and whole dairy products) ranging from 0 to 5 according to their position in the MD pyramid [38]. The total score ranges from 0 to 55, with higher values indicating greater adherence to the MD. A moderate alcohol intake, also typical of the MD, was not considered in this group of women since they were recommended not to drink alcohol during gestation. There were no women consuming alcohol during pregnancy. Therefore, the score considered for these analyses ranged from 0 to 50 points. 

### 2.9. Statistical Analysis 

Descriptive characteristics were summarized as mean (standard deviation (SD)) or frequencies (%) as appropriate. As initially designed [28], the primary statistical analysis was conducted using the per-protocol procedure. We only included women who attended more than 75% of exercise sessions and had valid postpartum body composition data [39]. We performed one-way analysis of covariance (ANCOVA) adjusted for age and pre-pregnancy BMI to explore differences in postpartum body composition between the control and exercise groups. Multiple imputations were performed for the intention-to-treat analyses, and the ANCOVA models were replicated including all the women recruited for the project according to the Consolidated Standards of Reporting Trials (CONSORT) guidelines. 

The choice of covariates was based a priori on what was previously reported in the literature. Age and pre-pregnancy BMI have been previously associated with body composition in pregnant women [17]. 

We explored the exercise*MD adherence interaction effects on the postpartum body composition. Potential moderators were further investigated when the exercise*MD interaction term showed a *p* < 0.1 and graphically represented using the median-split of the MD adherence score. Additionally, moderation analyses were conducted using the PROCESS macro 3.1 [40] to provide greater resolution for clarifying interactions. PROCESS utilizes ordinary least squares regression analysis when predicting continuous variables (i.e., postpartum body composition outcomes) and a bias-corrected bootstrap method (i.e., 5000 bootstrapped samples) to estimate the conditional (moderated) effects. The Johnson–Neyman technique was used to test for significance along a continuum of moderator values (i.e., MD score) and delineate the slope of the relationship. In this regard, the technique highlights specific MD adherence score cut-points in which the direction of the effect between the exercise intervention on the postpartum body composition changes (i.e., passes from negative to positive effect). The moderation analyses were adjusted for age and pre-pregnancy BMI. All analyses were conducted using the Statistical Package for Social Sciences (IBM SPSS Statistics for Windows, version 22.0, Armonk, NY, USA) and the level of significance was set to *p* < 0.05.

## 3. Results

Of the 83 pregnant who met the per protocol criteria (Supplementary Figure S1), the mean (SD) age was 33.5 (4.3) years, and the BMI at the postpartum was 25.4 (4.2) kg/m^2^. Most of the women had University degrees (n = 55, 66%) and were married (n = 49, 59%). At postpartum, mean (SD) weight retention was 4.1 (5.6) kg with 20% (n = 16) of women having returned to their pre-pregnancy body weight (Table 1). Women attended to 86% of the sessions on average. No between-group differences were found at baseline (all, *p* > 0.05). 

The effects of exercise on postpartum body composition are shown in Table 2. Per-protocol analyses showed that participants in the exercise group had lower postpartum BMI (between-group difference (95% CI): 1.18 [0.17–2.18] kg/m^2^; *p* = 0.022) and gynecoid fat mass (between-group difference (95% CI): 431.2 [3.52–858.89] g; *p* = 0.048). Participants in the exercise group had lower but non-statistically significant total fat mass (between-group difference (95% CI): 1723.2 [−236.9 to 3683.2] g; *p* = 0.084). We performed an additional analysis adjusting for weeks between birth–postpartum assessments and parity, but results remained unchanged.

These differences were no longer significant in the intention-to-treat analyses (Supplementary Table S2). 

**Figure 1:**
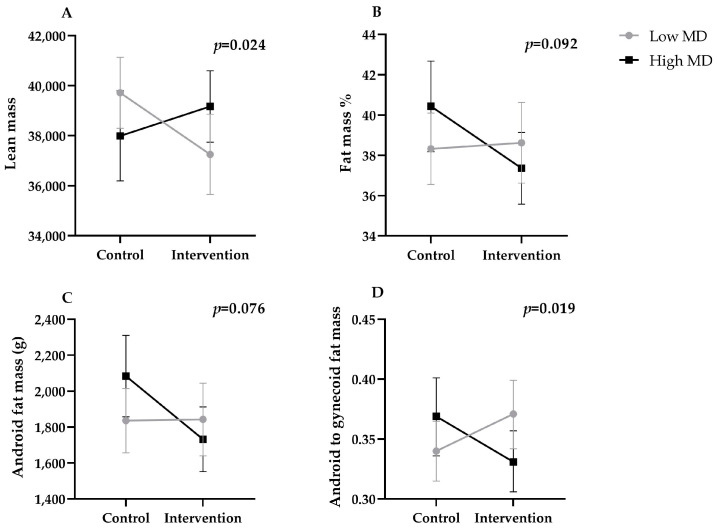
Differences in postpartum body composition outcomes according to exercise intervention and the degree of adherence to the Mediterranean diet (below median vs. above median). (**A**) Interaction between the exercise intervention and the MD adherence on the lean mass. (**B**) Interaction between the exercise intervention and the MD adherence over the fat mass percentage. (**C**) Interaction between the exercise intervention and the MD adherence over the android fat mass. (**D**) Interaction between the exercise intervention and the MD adherence over the android to gynecoid fat mass.

**Figure 2:**
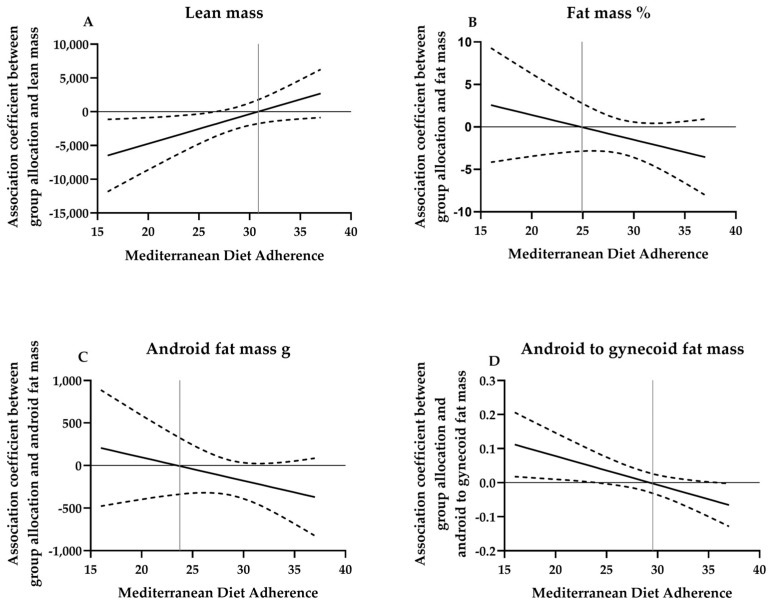
Regression slope estimate and 95% confidence interval for the effects of exercise on postpartum body composition outcomes as a function of the MD adherence. (**A**) Regression slope estimate and 95% confidence interval (dashed lines) for the effects of exercise on lean mass as a function of the MD adherence. (**B**) Regression slope estimate and 95% confidence interval (dashed lines) for the effects of exercise on fat mass as a function of the MD adherence. (**C**) Regression slope estimate and 95% confidence interval (dashed lines) for the effects of exercise on android fat mass as a function of the MD adherence. (**D**) Regression slope estimate and 95% confidence interval (dashed lines) for the effects of exercise on android to gynecoid fat mass as a function of the MD adherence.

The MD adherence during pregnancy was found to potentially moderate the effects of the exercise intervention on postpartum lean mass, fat mass percentage, android fat mass, and android-to-gynecoid fat mass (all *p*’s < 0.2). Those participants who received the exercise intervention and had a greater MD adherence showed higher lean mass, lower fat mass percentage, android fat mass, and android-to-gynecoid fat mass compared to participants who received the intervention and had a lower MD adherence (Figure 1). The effects of exercise on postpartum body composition outcomes as a function of the MD adherence score are shown in Figure 2. The Johnson–Neyman technique revealed that the direction of the effects changed at an MD Score of ~31 for lean mass, MD ~25 for fat mass, MD ~23 for android fat mass, and MD ~29 for android-to-gynecoid fat mass (percentiles 76, 20, 5, and 52 in the study sample, respectively). 

## 4. Discussion

Our results suggest that the effects of a concurrent exercise (aerobic + strength) program on postpartum body composition could be amplified by the adherence to a healthy dietary pattern (i.e., MD) during pregnancy. At postpartum, those women exercising and following an optimal MD pattern during pregnancy presented greater lean mass, lower percentage of fat mass, lower android fat mass and lower android-to-gynecoid fat mass compared to women exercising with a low MD adherence. Furthermore, we observed that the exercise effects were boosted with MD scores of >31 for lean mass, >25 for fat mass, >23 for android fat mass, and >29 for android-to-gynecoid fat mass. 

There is evidence suggesting that excessive PPWR contributes to long-term female overweight and obesity rates [3,26,41], which substantially increases the risk of diet-related chronic disorders [42]. PPWR at 6 weeks postpartum ranges from 3 to 7 kg [43] with an average weight retention of 3.7 kg at 6 weeks postpartum among European pregnant women [6], similar to the average weight retention that we found in the present study (i.e., 4.1 kg). Notwithstanding, only 20% of pregnant women at 6 weeks postpartum reached their baseline weight measured prior to pregnancy which is in agreement with previous evidence reporting a similar percentage (22%) [44]. 

In this context, it is well-known that diet and exercise are major components of most weight loss programs for the general population [41]. Concerning exercise, previous studies showed that neither physical activity [45] nor exercise [46] during pregnancy influenced BMI or body fat percentage during gestation and in postpartum body weight. Although Ruchat et al. [47] and Haakstad and Bo et al. [48] found that pregnant women who were randomized to an exercise intervention group during gestation retained less postpartum weight, no other postpartum body composition outcomes were assessed. On a per-protocol basis (when only considering those women with >75% attendance), we found a significant reduction in BMI and gynecoid fat mass in the exercise compared with the control group. These associations were no longer significant in the intention-to-treat analyses (after multiple imputation of the data). This may be partially explained by differences in the sample size, but also by the fact that women included in the intention-to-treat analyses did not attend 75% of the exercise intervention programme, or even dropped out of the study. In fact, the average attendance was 86% in the per-protocol sample and 75% when including all women (intention to treat). Therefore, it seems plausible that a mitigation of the effects of the exercise intervention on postpartum body composition might be observed on an intention-to-treat basis analysis. 

We did not find between-groups differences on postpartum body weight without accounting for the moderation effect of diet. However, according to a systematic review [26], exercise alone is not sufficient to promote weight loss in women after childbirth. Interventions including diet and exercise appear to be more effective in promoting postpartum body weight and fat mass loss [25] than exercise alone [16]. Exercise helps burn calories, build muscle, and improve metabolism. Meanwhile, diet provides the necessary nutrients for energy, muscle repair, and overall health. The combination optimizes fat loss, preserves lean muscle mass, and enhances the effectiveness of each component. Interestingly, those participants who received the exercise intervention and had an optimal MD adherence (above median) did not show differences in postpartum body weight but showed a higher lean mass, a lower percentage of fat mass, and a healthier distribution of fat mass (i.e., lower android fat mass and lower android-to-gynecoid fat mass). As a consequence of the increase in one body composition component (i.e., lean mass) and the decrease in another (i.e., fat mass), the total weight might have remained stable between exercise and control groups. However, there were positive changes in postpartum body composition outcomes, which might be clinically meaningful as previously stated [49]. In addition, we observed that adhering to a MD pattern might be crucial to either observe or boost these effects of the exercise on the postpartum body composition. This adherence to MD ought to be above 23 (percentile 14.5 in the study sample) for android fat mass, above 25 (percentile 20.5) for fat mass percentage, above 29 (percentile 51.8) for android-to-gynecoid fat mass, and above 31 (percentile 75.9) for lean mass. 

Therefore, our findings show the potential role of the diet on the effects of exercise on postpartum body composition. However, this should be further tested by combining diet and exercise interventions in randomized controlled trials. 

The results of the present study should be considered in light of some limitations. Firstly, this is quasi-experimental study, which is a limitation since women were not purely randomized. Secondly, our sample size might be limited, and larger trials should contrast our findings. Thirdly, body composition was only assessed after 6 weeks postpartum, thus, longer follow-ups could provide additional useful information. Regarding strengths, the measurement tool employed to assess body composition (i.e., DXA) is widely valid and reliable, which guarantees the quality of the data. Furthermore, a detailed definition of the dietary habits and a valid assessment of the MD diet adherence was employed. In addition, the exercise group followed a novel concurrent exercise that combines aerobic + strength training, which has been proved as the most useful protocol to improve cardiometabolic status during pregnancy [50].

## 5. Conclusions

Overall, our results suggest that the exercise effects on postpartum body composition might be enhanced by following a healthy dietary pattern (i.e., MD) during pregnancy. Specifically, those participants who received the exercise intervention and had an optimal MD adherence showed greater lean mass, lower fat mass and a healthier distribution of body fat. Further randomized controlled trials are needed to examine the benefits of a combined exercise and diet intervention during pregnancy to reduce adiposity and preserve lean mass beyond pregnancy. The inclusion of dietary advice within exercise training programs should be considered in future projects.

## Figures and Tables

**Table 1:** Anthropometric and sociodemographic characteristics of the study sample.

Variable	Total Women (n = 83)	Control (n = 40)	Exercise (n = 43)
Age (years)	33.5 (4.3)	33.8 (4.3)	33.3 (4.3)
Pre-pregnancy body mass index (kg/m^2^)	23.7 (3.9)	23.2 (3.3)	24.4 (4.0)
Total physical activity (min/day) (n = 36 vs. 41)	427.0 (94.2)	437.6 (96.8)	417.7 (92.0)
Moderate–vigorous physical activity (min/day) (n = 36 vs. 41)	39.2 (21.7)	37.8 (23.4)	40.5 (20.3)
Percentage of attendance *			86.3 (6.5)
Postpartum body composition			
Body weight (kg) (n = 37 vs. 42)	68.2 (10.7)	68.0 (70.8)	68.4 (10.7)
Height (cm) (n = 37 vs. 42)	163.6 (5.6)	163.5 (5.5)	163.7 (5.7)
Postpartum weight retention (kg) (pp-pre) (n = 37 vs. 42)	4.1 (5.6)	5.8 (4.6)	2.6 (5.9)
Body mass index (kg/m^2^) (n = 37 vs. 42)	25.4 (4.2)	25.5 (3.99)	24.4 (4.0)
Total lean mass (g)	38,665.1 (4514.6)	38,544.8 (4780.2)	38,776.9 (4306.8)
Total fat mass (g)	26,138.6 (7257.6)	25,993.0 (6916.4)	26,273.9 (7640.5)
Total fat mass (%)	38.5 (5.5)	38.5 (5.3)	38.5 (5.8)
Total android fat mass (g)	1855.1 (701.0)	1834.4 (637.8)	1874.5 (762.1)
Total gynecoid fat mass (g)	5218.7 (1280.9)	5287.8 (1215.6)	5154.3 (1350.0)
Visceral fat (g)	365.3 (152.7)	363.3 (152.3)	367.1 (154.8)
Gynecoid to total fat mass ratio	0.202 (0.02)	0.206 (0.02)	0.198 (0.02)
Android to gynecoid fat mass	0.350 (0.08)	0.343 (0.08)	0.357 (0.08)
Mediterranean Diet adherence (0–50)	29.1 (3.9)	28.4 (4.0)	29.7 (3.7)
Educational status			
University degree	55 (66.3)	29 (72.5)	26 (60.5)
No university degree	28 (33.7)	11 (27.5)	17 (39.5)
Marital status			
Married	49 (59.0)	24 (60.0)	25 (58.1)
Single/divorced/widow	34 (41.0)	16 (40.0)	18 (41.9)
Type of breastfeeding (n = 81)			
Exclusive (only breast)	55 (67.9)	23 (60.5)	32 (74.4)
Mixed (breast and formula milk)	16 (19.8)	10 (26.3)	6 (14.0)
Artificial (only formula milk)	10 (12.3)	5 (13.2)	5 (11.6)
Smoking habit (yes, n (%))	6 (7.2)	5 (12.5)	1 (2.3)
Alcohol intake (yes, n (%))	0 (0)	0 (0)	0 (0)
Women returning to pre-pregnancy weight (yes, n (%)) ^a^ (n = 37 vs. 42)	16 (20.3)	1 (2.7)	15 (35.7)

Values shown as mean (standard deviation) unless otherwise indicated. SD, standard deviation. PPWR, postpartum weight retention. * When considering women on an intention to treat basis, the average percentage of attendance was 75.3%. ^a^ Women who weighed the same or less compared to their weight prior to pregnancy.

**Table 2:** Influence of the exercise program on postpartum body composition.

Variable	Control (n = 40)	Exercise (n = 43)	Between-Group Difference (B) (95% CI)	*p*
Per-protocol basis *				
Body weight (kg) (n = 37 vs. 42)	69.59 (1.05)	67.33 (0.99)	2.26 (−0.63 to 5.15)	0.124
Body mass index (kg/m^2^) (n = 37 vs. 42)	26.07 (0.36)	24.89 (0.35)	1.18 (0.17–2.18)	0.022
Total lean mass (g)	39,054.49 (568.19)	38,302.81 (547.72)	751.68 (−830.81 to 2334.17)	0.347
Total fat mass (g)	27,031.30 (703.76)	25,308.14 (678.40)	1723.16 (−236.91 to 3683.22)	0.084
Total fat mass (%)	39.14 (0.70)	37.95 (0.67)	1.19 (0–0.75 to 3.14)	0.226
Total Android fat mass (g)	1931.42 (70.77)	1784.20 (68.22)	147.23 (−49.89 to 344.34)	0.141
Total gynecoid fat mass (g)	5442.07 (153.56)	5010.86 (148.03)	431.21 (3.52–858.89)	0.048
Visceral fat (g)	381.16 (18.1)	350.5 (17.5)	30.65 (−19.87 to 81.17)	0.231
Gynecoid to total fat mass	0.204 (0.002)	0.200 (0.002)	0.004 (−0.002 to 0.010)	0.196
Android to gynecoid fat mass	0.351 (0.010)	0.349 (0.010)	0.002 (−0.027 to 0.030)	0.897

Values shown as mean (standard error). SE, standard error. Model adjusted by age and pre-pregnancy body mass index (kg/m^2^). * The average percentage of attendance was 86%.

## Data Availability

The datasets used and/or analyzed during the current study are available from the corresponding author on reasonable request.

## Supplementary Materials

The following supporting information can be downloaded at: https://www.mdpi.com/article/10.3390/nu15204413/s1. Supplementary Table S1: Inclusion and exclusion criteria in the GESTAFIT project; Supplementary Figure S1: Flow diagram of the study participants; Supplementary Table S2: Influence of the exercise program on postpartum body composition; File S1: Lectures provided to pregnant women.
