# Supplementary material for: Impact of Exercise Intervention Combined with Optimal Mediterranean Diet Adherence during Pregnancy on Postpartum Body Composition: A Quasi-Experimental Study—The GESTAFIT Project
Source: Nutrients. 2023 Oct 18;15(20):4413. doi: 10.3390/nu15204413 (PMC10609918; doi:10.3390/nu15204413)
Supplement: Supplementary file 1 [file nutrients-15-04413-s001.zip › nutrients-2653524-supplementary.pdf]

Supplementary Material

Supplement to: “**Impact of Exercise Intervention Combined with Optimal Mediterranean Diet Adherence during Pregnancy on Postpartum Body Composition: A Quasi-Experimental Study—The GESTAFIT Project”**

Table of contents

File S1. Lectures provided to pregnant women

Supplementary Figure S1. Flow diagram of the study participants.

Supplementary Table S1. Inclusion and exclusion criteria in the GESTAFIT project.

Supplementary Table S2. Influence of the exercise program on postpartum body composition.

File S1. Lectures provided to pregnant women

During the duration of the intervention, the research team gave 7 lectures to pregnant women from both groups (exercise and control group) about: 1) the benefits of physical exercise for a better pregnancy, prevention and treatment of cardiovascular diseases and excessive weight gain; 2) ergonomic advises, exercises to perform at home and strategies to increase their daily physical activity levels; 3) the benefits of the Mediterranean Diet and nutritional education during pregnancy; 4) how to avoid toxics and chemicals during the pregnancy and breastfeeding; 5) pregnancy, postpartum and sex; 6) physical and mental preparation for the labour, what to expect; 7) nutritional education towards breastfeeding. We also used these conferences to maintain control group fidelity until the end of the program.

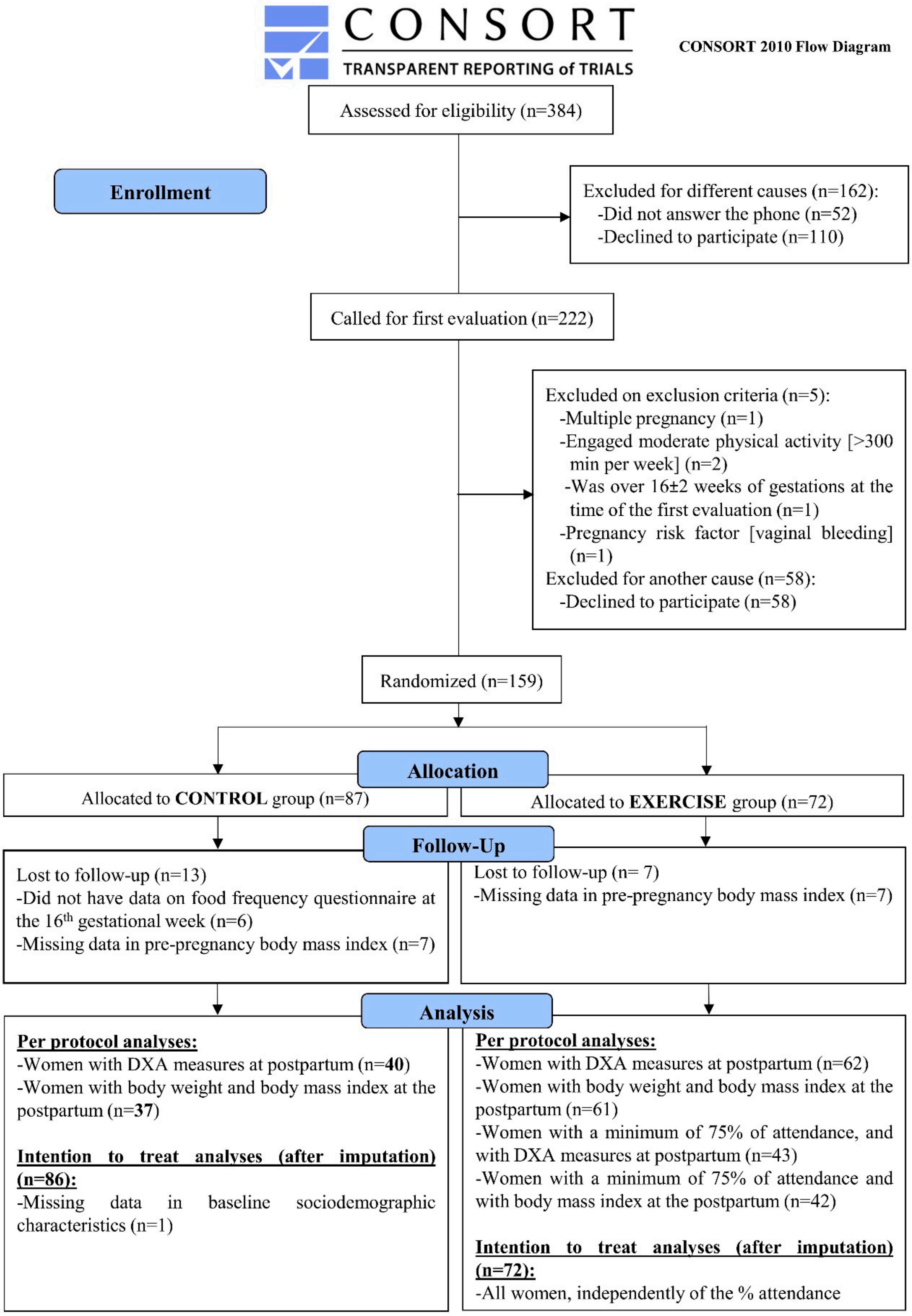

Supplementary Figure S1. Flow diagram of study participants.

Supplementary Table S1. Inclusion and exclusion criteria in the GESTAFIT project.

**Inclusion criteria**
- Pregnant women aged 25-40 years old with a normal pregnancy course.
- Answering "no" to all questions on the PARmed-X for pregnancy.
- Being able to walk without assistance.
- Being able to read and write properly.
- Informed consent: Being capable and willing to provide written consent.
**Exclusion criteria**
- Having acute or terminal illness.
- Having malnutrition.
Being unable to conduct tests for assessing physical fitness or exercise during pregnancy.
- Having pregnancy risk factors (such as hypertension, type 2 diabetes, etc.).
- Having a multiple pregnancy.
- Having chromosopathy or foetal malformations.
- Having uterine growth restriction.
- Having foetal death.
- Having upper or lower extremity fracture in the past 3 months.
- Suffering neuromuscular disease or presence of drugs affecting neuromuscular function.
- Being registered in another exercise program.
- Performing more than 300 minutes of at least moderate physical activity per week.
-Being engaged in another physical exercise program
- Being unwilling either to complete the study requirements or to be randomized into the control or intervention group.

Supplementary Table S2. Influence of the exercise program on postpartum body composition.

	Control (n = 86)	Exercise (n = 72)	Between-Group Difference (B) (95% CI)	*P*
Intention to treat*				
Body weight (kg)	68.7 (0.7)	67.8 (0.7)	0.845 (-1.078 to 2.768)	0.387
Body mass index (kg/m^2)	25.9 (0.2)	25.4 (0.3)	0.516 (-0.194 to 1.225)	0.153
Total lean mass (g)	38779.5 (365.8)	39071.6 (400.0)	-292.078 (-1365.919 to 781.763)	0.592
Total fat mass (g)	24438.4 (1782.8)	25193.2 (1949.5)	-754.845 (-5988.664 to 4478.974)	0.776
Total fat mass (%)	35.8 (2.0)	37.8 (2.2)	-2.093 (-8.112 to 3.926)	0.493
Total Android fat mass (g)	1693.3 (260.7)	1762.2 (285.1)	-68.968 (-834.416 to 696.480)	0.859
Total gynecoid fat mass (g)	5603.7 (196.4)	5220.6 (214.7)	383.149 (-193.279 to 959.577)	0.191
Visceral fat (g)	371.6 (12.5)	374.7 (13.6)	-3.082 (-39.678 to 33.514)	0.868
Gynecoid to total fat mass	0.216 (0.058)	0.185 (0.063)	0.031 (-0.139 to 0.201)	0.718
Android to gynecoid fat mass	0.143 (0.090)	0.323 (1.0)	-0.180 (-0.445 to 0.085)	0.182

Values shown as mean (standard error). SE, standard error. Model adjusted for age and pre-pregnancy body mass index (kg/m^2). * The average percentage of attendance was 75%
